# Treatment outcomes of a Swiss non-infectious paediatric uveitis cohort: retrospective study over ten years

**DOI:** 10.1186/s12348-025-00458-w

**Published:** 2025-05-08

**Authors:** Jeanne Martine Gunzinger, Seraina Palmer Sarott, Fabio Meier, Christian Böni, Alice Kitay, Brigitte Simonsz-Tóth, Christina Gerth-Kahlert

**Affiliations:** 1 Department of Ophthalmology, University Hospital Zurich, University of Zurich, Frauenklinikstrasse 24, Zurich, 8091 Switzerland; 2 Department of Paediatric Rheumatology, University Children’s Hospital Zurich, Zurich, Switzerland; 3 Augenzentrum Witikon, Zurich, Switzerland; 4 Augenarztpraxis Bremgarten, Bremgarten, Switzerland

**Keywords:** Children, Paediatric, Uveitis, TNF alpha inhibitor, Adalimumab, Methotrexate

## Abstract

**Introduction:**

Paediatric uveitis treatment recommendations suggest a step-up treatment approach starting with topical treatment, followed by antimetabolites and thereafter biologics. With this study, we are investigating the safety and efficacy of the current treatment approach in a large cohort.

**Material and methods:**

Single center retrospective study. Patients with non-infectious uveitis under the age of 18 years at first presentation, between January 2012 and June 2022, were eligible for inclusion. Data extracted from the electronic health records included age at first presentation, sex, involved eye segment, visual acuity (VA), complications, associated systemic disease, treatments, and number of consultations. Cases were grouped according to their final treatment regime (topical only, methotrexate, TNF alpha inhibitor, other). VA outcome, treatment response, adverse events, and frequency of consultations were evaluated. The study was approved by the local ethics committee.

**Results:**

64 non-infectious paediatric uveitis cases were included. Age at first diagnosis ranged from 2 to 17 years, with a two-peak distribution, 52% were male. Anterior uveitis was the most common presentation, followed by intermediate uveitis, posterior uveitis, and panuveitis. Topical treatment achieved remission in 23%, anti-metabolites in 12%, and escalation to TNF alpha inhibitors in 30%. Alternative treatments or observation only were documented in 16% and 17%, respectively. Median duration from first presentation to the start of anti-metabolite or TNF alpha inhibitor were 115 days and 269 days, respectively. There was a median of eight consultations during the first year of follow up. Frequency of consultations during the first year increased with every treatment escalation. VA outcome did not differ between the different treatment groups.

**Conclusion:**

The step-up treatment approach shows a safe profile in regards to VA outcome. Methotrexate presents a high rate of treatment failure and adverse effects. Adalimumab and infliximab are effective and safe. Timely treatment escalation might lower treatment burden for affected children, their families, and health care providers.

## Introduction

Uveitis is a sight-threatening disease and estimated to be the cause of blindness in 3–10% [1–5]. The incidence of paediatric uveitis is estimated between 4.9 and 14 per 100’000 per year; paediatric uveitis accounts for about 5–10% of all uveitis cases [6–9]. Uveitis is usually classified by the primary site of inflammation, according to the standardization of uveitis nomenclature (SUN) [10, 11]: anterior, intermediate, posterior, or panuveitis. Any form of uveitis can be associated with sight-threatening complications such as glaucoma, cataract, band keratopathy, hypotony, and macular oedema [5, 12]. In children, complications are common (35.5–67%), with one third already present at time of diagnosis [8, 13–17]. The main goal in treating any uveitis is control of inflammation and preservation of vision, as well as minimize adverse effects of treatments [18–21]. Current practice is a step-up treatment approach: Corticosteroids are used as first line medication due to their rapid response and control of inflammation [19, 22, 23]. Systemic steroids are mainly used in sight-threatening disease [24, 25]. Consensus based recommendations for juvenile idiopathic arthritis (JIA) associated uveitis and idiopathic chronic anterior uveitis advise an escalation to methotrexate if quiescence cannot be achieved with a maximum of 3 drops of topical dexamethasone 0.1% or prednisolone acetate 1% per day [19, 25–27]. In case of inefficacy of - or intolerance to – methotrexate, escalation to the tumor necrosis factor (TNF) alpha inhibitor adalimumab is suggested, with infliximab as an alternative in some recommendations [19, 25, 27–29].

This paper presents real word data on treatment and outcome of children with non-infectious uveitis under the care of a large tertiary center over a ten-year period.

## Methods

This is a single center retrospective study. Electronic health records (EHRs) were searched automatically. Search criteria included first presentation between 01.01.2012 and 30.06.2022, diagnosis of uveitis and under 18 years of age at first presentation. EHRs were manually reviewed for patients with non-infectious uveitis and positive informed consent. Demographic and ocular data extracted included age at first presentation, year of first presentation, sex, affected segment, and laterality, habitual distance visual acuity (VA, Snellen, decimal, as measured in clinic), intraocular pressure (IOP), and ocular complications. In our centre, children presenting with uveitis are routinely seen by a paediatric rheumatologists (and other paediatric specialists if deemed necessary) for a complete clinical exam and are taken bloods to screen for possible associated diseases. Extraocular data extracted from EHRs included associated systemic disease, antinuclear antibody (ANA) status, and human leukocyte antigen (HLA) B27 status. Further, treatments such as eye drops, oral or parenteral medication, and ophthalmic interventions (intravitreal injections, retinal lasercoagulation, surgery) were recorded. Follow up EHR entries until 31.12.2023 were used.

Following subgroups were compared: Patients who received (group 1) topical treatment only, (group 2) methotrexate, and (group 3) TNF alpha inhibitors. Reason for treatment escalation was recorded (treatment failure or adverse effects). Baseline characteristics, visual outcome, and number of consultations in the first year of follow up were compared. Risk factors for development of complications and poor visual outcome were evaluated.

Statistics was performed using IBM SPSS Statistics. Appropriate tests were used for comparison of paired samples (Wilcoxon signed rank test), correlations (Pearson correlation and odds ratio) as well as correction for multiple comparison where needed (Bonferroni correction). Significance level was set at *p* < 0.05.

Local ethics committee approved this study (Institutional review board of Swiss Ethics/BASEC No. 2023 − 00439). The Tenets of the Declaration of Helsinki were followed.

## Results

### Baseline demographics

**Fig. 1:**
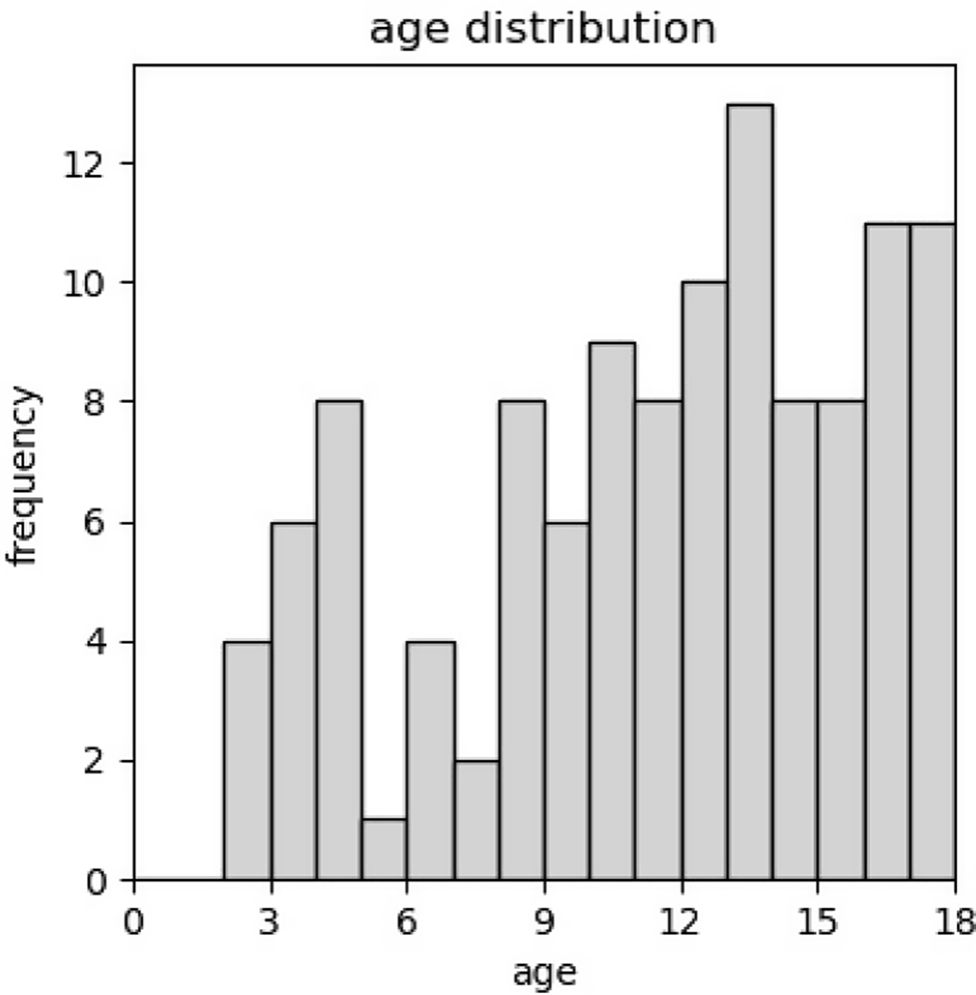
Histogram of age distribution at diagnosis. There is a two-peaked distribution. The first peak is in pre-school and then a second one in teenage years with a steady increase towards later adolescence

A total of 133 paediatric uveitis patients with first presentation at our tertiary center between January 2012 and June 2022 were identified. Thereof, 22 negated consent and 15 were not reachable. Out of the 96 eligible patients, 64 had non-infectious paediatric uveitis (53/64 presented bilateral uveitis during their disease course, with a total of 117 eyes affected). 52% were male. Mean age at initial referral was 11.7 years (range 2.0–17.6 years). The distribution shows one peak in preschool age and a steady incline from 8 to 17 years of age as shown in Fig. 1. Mean VA at first presentation was 0.79, ranging from hand movement to 1.6. Most presented with anterior uveitis (48%), followed by intermediate uveitis (30%), mixed anterior-intermediate uveitis (9%), posterior uveitis (9%) and panuveitis (3%). ANA titer were positive in 23 of 50 tested patients. In contrast, HLA B27 titer was positive in 6/37 tested patients. 

Most cases were of unknown aetiology (56%). Four patients (6%) presented ANA + chronic anterior uveitis without systemic disease and two patients (3%) presented with HLA B27 typicial anterior uveitis without associated systemic disease. Most common associated systemic disease was JIA (13%), others included tubulointerstitial nephritis and uveitis, systemic lupus erythematodes, ankylosing spondylitis, familial Mediterranean fever, multiple sclerosis and Susac syndrome. At least one ocular complication was documented in 39 eyes of 23 patients at first presentation (band kerathopathy, synechiae, cataract, disc swelling, disc atrophy, macular edema, epiretinal membrane, macular atrophy, or vitreous hemorrhage), detailed data is presented in Table 1. Posterior and panuveitis presented the highest rates of complications at first presentation. Median duration of follow up was 763 days (i.e. 2 years and one month; interquartile range 100 to 1726 days).

**Table 1:** Ocular complications at first presentation and new onset during follow up

	At first presentation *n* = eyes	New onset during follow up *n* = eyes
**Anterior (51 eyes of 31 patients)**		
- Band keratopathy - Posterior synechiae - Cataract - Optic disc swelling - Macular edema - Glaucoma **- ANY**	− 2 − 6 − 2 − 2 − 1 − 0 **− 8**	− 0 − 0 − 1 − 0 − 0 − 1 **− 1**
**Anterior – intermediate (12 eyes of 6 patients)**		
- Posterior synechiae - Optic disc swelling - Macular edema - Vitreous haemorrhage **- ANY**	− 2 − 5 − 2 − 0 **− 6**	− 0 − 2 − 0 − 1 **− 3**
**Intermediate (38 eyes of 19 patients)**		
- Band keratopathy - Cataract - Optic disc swelling - Macular edema - Epiretinal membrane - Macular atrophy - Retinal neovascularisations - Vitreous haemorrhage **- ANY**	− 0 − 3 − 7 − 0 − 0 − 2 − 0 − 1 **− 13**	− 4 − 1 − 1 − 1 − 1 − 0 − 3 − 1 **− 8**
**Posterior (12 eyes of 6 patients)**		
- Band keratopathy - Optic disc swelling - Macular edema - Macular atrophy - Disc atrophy - Vitreous haemorrhage **- ANY**	− 2 − 2 − 0 − 4 − 2 − 0 **− 8**	− 0 − 1 − 1 − 1 − 2 − 1 **− 2**
**Panuveitis (4 eyes of 2 patients)**		
- Band keratopathy - Posterior synechiae - Cataract - Optic disc swelling - Macular edema - Epiretinal membrane - Macular atrophy - Vitreous haemorrhage **- ANY**	− 2 − 4 − 2 − 2 − 0 − 3 − 1 − 0 **− 4**	− 0 − 0 − 0 − 0 − 1 − 0 − 0 − 1 **− 1**

### Treatment analysis

**Fig. 2:**
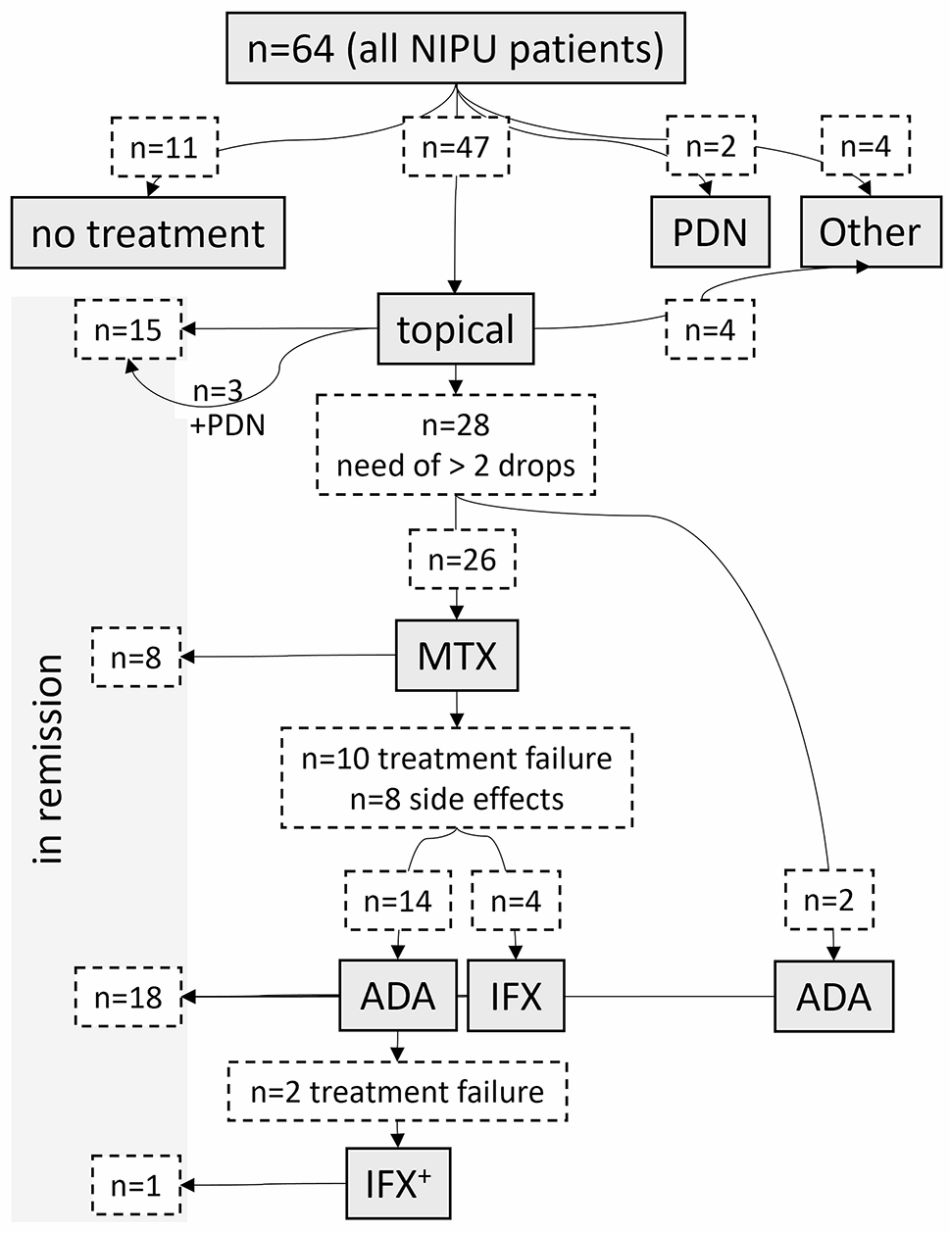
Flow chart of treatment pathway. Most patients went through a step-up treatment ladder, first using topical steroid eye drops (prednisolone acetate 1% or dexamethasone 0.1%), followed by methotrexate, followed by TNF inhibitor adalimumab. In some cases (mostly patients seen before 2017) infliximab was used as a first line biologic. In two cases, infiliximab was used as alternative treatment in case of adalimumab refractive uveitis. *NIPU*,* non-infectious paediatric uveitis; PDN*,* oral or intravenous prednisone; MTX*,* methotrexate; ADA*,* adalimumab; IFX*,* infliximab;*^*+*^*One patient lost to follow up before treatment response could be evaluated*

Patients were usually treated with a step-up treatment approach as summarized in see Fig. 2.

Topical treatment (prednisolone acetate 1% or dexamethasone 0.1%) was initiated in 47/64 (73%). This led to remission in 19% of the patients. Treatment could be ceased within three months without recurrence in 13% of these patients. An additional short course of systemic cortisone (but no further systemic treatment) was used in 5% to achieve remission. A steroid response to topical steroids with raised IOP necessitating temporary use of pressure lowering eye drops was recorded in eight patients.

Methotrexate was introduced in 26/64 (41%) of patients. Remission was achieved in eight patients (31%) (with a maximum of two drops of topical steroids daily). Despite treatment, ten patients (38%) had insufficient control of their uveitis. Substantial side effects such as nausea and/or abnormal liver function tests were documented in an additional eight patients on methotrexate treatment (31%).

Escalation to TNF alpha inhibitors adalimumab or infliximab were instigated in 18/64 (28%). An additional two patients (3%) were started on a TNF alpha inhibitor without a prior treatment with methotrexate. Between 2012 and 2016, adalimumab and infliximab were used equally frequent as first line biologic. From 2017 onwards, adalimumab was the first line TNF alpha inhibitor except for one patient. Adalimumab in combination with methotrexate lead to remission in 7 out of 7 patients. Adalimumab monotherapy lead to remission in 7 out of 9 patients. The other two were both were switched to infliximab.

Infliximab (with methotrexate or other antimetabolite) led to remission in five out of six patients; one was lost to follow up before treatment response could be evaluated. In regards to adverse effects, one patient developed a skin abscess while on adalimumab; however, the installed treatment could be continued. No adverse reactions were recorded from infliximab in any patient.

Immunmodulating medications other than methotrexate and TNF alpha inhibitors were used in 8/64 (13%). This was usually guided by their associated systemic disease (Leflunomide for JIA, Privigen^®^ for Susac syndrome, cyclophosphamide for systemic lupus erythematodes, Tecfidera^®^ for demyelinating disease, sirolimus for immunodeficiency syndrome, colchicine for autoimmune fever). A brief course of standalone systemic cortisone was used in 2/64 (3%). No treatment was introduced in 11/64 (17%) of patients.

Topical treatment was usually started at first visit (median 0 days). Median duration from initial referral to start methotrexate was 115 days (interquartile range 68 to 243 days). Median duration to start TNF alpha inhibitor (counted from first visit) was 269 days (interquartile range 129 to 515 days).

### VA outcome

**Fig. 3:**
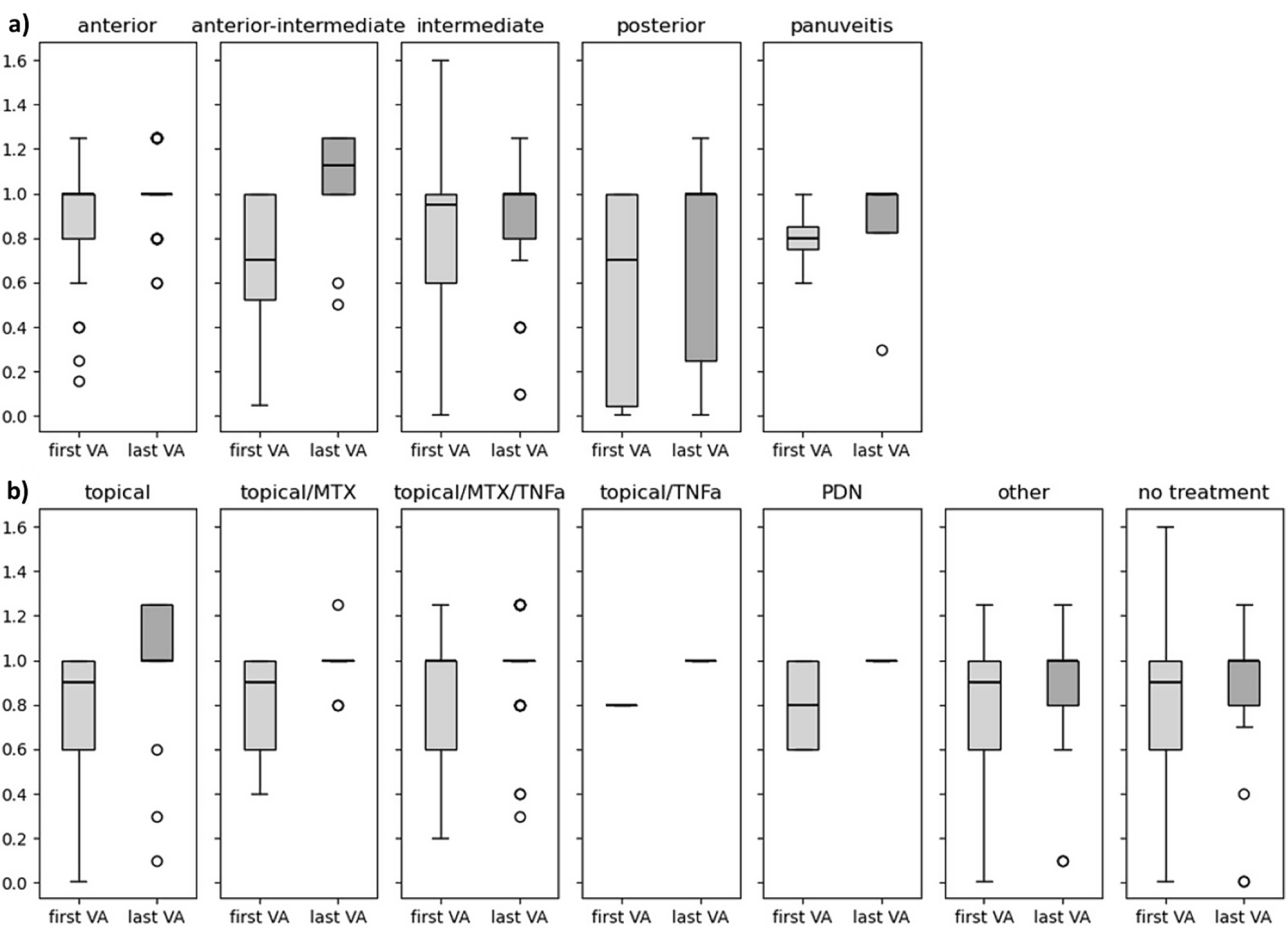
Visual acuity (VA) at first visit and last follow up. Mean VA outcome did not differ between the primarily affected segment (**a**) nor in regards to different treatment groups (**b**). There is a notably higher variability in posterior uveitis. *Topical*,* prednisolone acetate 1% or dexamethasone 0.1%*,* MTX*,* methotrexate; TNFa*,* tumor necrosis factor alpha inhibitors adalimumab or infliximab; PDN*,* oral or intravenous prednisone*

Overall VA in affected eyes improved significantly from mean 0.79 (SD ± 0.33) to mean 0.94 (SD ± 0.27) from first to last consultation (*p* < 0.001). Out of 117 eyes, 5 had a VA below 0.2 at last follow up. VA at first presentation correlates significantly with VA outcome (*p* < 0.001). Mean VA outcome did not differ between the affected segments, but scattering was considerably higher in posterior uveitis compared to the other segments (Fig. 3a). VA at first presentation and VA outcome did not differ between the different treatment groups (Fig. 3b).

Five patients were diagnosed with amblyopia in association with their uveitis. Two (aged 8 and 9 years) were assumed to have bilateral mixed cause of their amblyopia with a combination of ametropia and deprivation secondary to their uveitis; three (aged 3, 4, and 6 years) were assumed to have deprivation amblyopia primarily due to their uveitis or complications thereof (two had a significant cataract in one eye at first presentation). The two older patients with mixed aetiology - both with bilateral deprivation - showed only minimal VA improvement over time despite uveitis and amblyopia treatment (from mean VA of 0.47 at first presentation to 0.57 at last follow up). The three younger patients - two received occlusion therapy due to unilateral amblyopia - showed pronounced VA improvement in the affected eyes (mean 0.31 to 0.95).

### Complications and procedures during follow up

Complications during follow up were documented in 20% of patients (band keratopathy, cataract, disc swelling, macular edema, epiretinal membrane, retinal neovascularization, and vitreous hemorrhage), see Table 1. Patients with complications at first presentation developed substantially more additional complications during follow up compared to patients without any complication initially (39% vs. 10%, odds ratio 5.9). Patient with anterior – intermediate or intermediate uveitis had the highest rate of new onset complications during follow up. Patients with complications at first consultation were more often treated with oral or intravenous prednisone (odds ratio 4.0) and/or a steroid-sparing immunosuppressant (odds ratio 1.8).

Three patients had cataract surgery (all with artificial intraocular lens implantation), one had glaucoma surgery (XEN^®^ gel stent with mitomycin C), five patients received retinal laser, and two patients had intravitreal injections (one received intravitreal triamcinolone and the other intravitreal bevacizumab).

### Consultation frequency

**Fig. 4:**
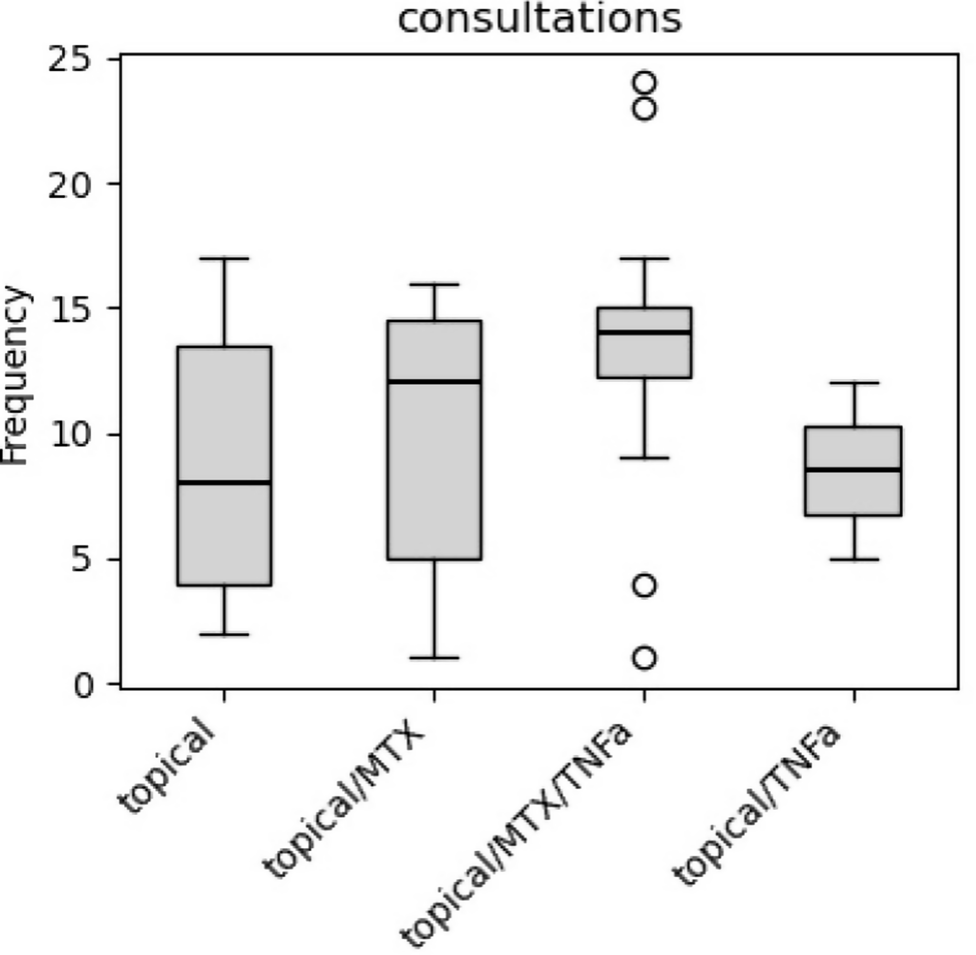
Frequency of consultations during the first year of follow up. Number of consultations tended to be higher with every step of treatment escalation. *Topical*,* prednisolone acetate 1% or dexamethasone 0.1%; MTX*,* methotrexate; TNFa*,* tumor necrosis factor alpha inhibitors adalimumab or infliximab*

During the first year of follow up, patients had a median of 8 consultations (interquartile range 3 to 14 consultations) at our center. Number of consultations in the first year increased with each necessary treatment escalation, from median 8 consultations in patients in remission on topical treatment, to 12.5 consultations in patients in remission on methotrexate and 14 consultations for patients escalated to TNF alpha inhibitors (See Fig. 4).

## Discussion

Our study presents retrospective real world data of non-infectious paediatric uveitis patients. The majority of patients suffered from anterior uveitis, presented bilateral disease, had an unknown aetiology; one third presented complications at first visit - all in accordance with the literature [22, 30, 31]. Thus, this cohort is representable for many non-infectious paediatric uveitis clinics.

A recent study has demonstrated poor compliance in regards to eye drop applications in paediatric uveitis [32]. In our sample, nearly a quarter of patients reached remission on topical treatment alone. Eye drops compliance might be better in the initial phases compared to long-term treatments, but we have not systematically assessed compliance rate. Topical steroids can be associated with side effects, mainly cataracts and glaucoma. Cataract development seems to be dose dependent and very rare with a frequency of less than 3 drops per day [33]. The cataracts reported in our cohort were assumed to be mostly due to their uveitis activity. Risk of steroid-induced intraocular hypertension in children is higher than in adults and also dose dependent [34]. In our cohort, one sixth of patients on topical steroids developed an IOP increase necessitating temporal use of pressure lowering drops and either reducing or stopping the steroid eye drops until IOP normalised. This findings underlines the recommendation to follow up children not only for uveitis activity but also for IOP to prevent glaucomatous damage [30, 35].

Methotrexate is recommended as first line treatment for non-infectious uveitis, but the level of evidence is only moderate, and it is well known for its adverse side effects [36–41]. Our sample support this, as 38% showed treatment failure and an additional 31% suffered non-manageable side effects and emphasizes the challenges of treating paediatric uveitis with methotrexate.

Adalimumab has proven to be effective for treatment of pediatric non-infectious uveitis and is approved by many authorities for this indication [13, 42–47]. Our data confirms the efficacy of adalimumab in paediatric non-infectious uveitis with a very high remission rate. Adalimumab has an overall good safety profile, with infections being the most reported adverse events [48–50]. One of the patients in our cohort had developed a skin abscess while on adalimumab. No serious adverse reactions were recorded. Since the SYCAMORE and ADJUVITE trials, European consensus recommendations list adalimumab as the first line biologic for paediatric non-infectious uveitis [19, 26]. Timely accessibility to adalimumab can be hindered by local health authorities (the Swiss government for example requires prior authorization for adalimumab and, depending on the insurance company, often requiring a trial of methotrexate first). It’s use is further limited for very young children, as it is only approved for children aged four years and older.

Infliximab is a valid alternative treatment in case of treatment failure of adalimumab [51]. Our cohort includes patients treated before the publication of the adalimumab RCTs, and therefore some patients had infliximab as a first line biologic, all of which achieved remission. Only two patients were switched from adalimumab to infliximab, of which one achieved remission and the other was lost to follow up before effect could be evaluated. Alternative suggested treatment, although with limited evidence, include tocilizumab, rituximab, and janus kinase (JAK) inhibitors [26].

Combination of adalimumab with methotrexate seems preferable then standalone treatment, as the prevelance of adalimumab antibodies is inversely related to the dosing of simultaneous methotrexate [52]. Nevertheless, in case of intolerance to methotrexate, adalimumab monotherapy is an effective treatment option [45]. This is supported by our sample with a high remission rate on adalimumab monotherapy. Our cohort was not routinely screened for antibodies, we can therefore not evaluate if there was a correlation between antibody levels and treatment failure.

The VA outcome did not differ between the different treatment groups, supporting the safety of the step up treatment approach, even if it took significantly longer to establish the treatment with every escalation step (median 0 days vs. 115 days vs. 269 days). Higher powered studies would be needed for more detailed results regarding the complication rate in the different sub groups. We do like to highlight the higher complication rate in intermediate uveitis compared to anterior uveitis, which might indicate a undertreatment due to a generally less aggressive approach in milder intermediate uveitis. Five patients were diagnosed with amblyopia already at the beginning of their uveitis treatment, increasing the risk for vision loss [53]. One study even identified amblyopia as the leading cause for vision loss in paediatric uveitis patients under 8 years old [53]. Combined uveitis and amblyopia treatment was very effective in the younger patients in our sample. We did not record any development of amblyopia during follow up in our cohort, but children with uveitis are at risk of amblyopia and should be monitored as they might require amblyopia treatment in addition to their uveitis treatment [35, 54–56]. The possibility of amblyopia as a reason for vision reduction in children with uveitis should always be evaluated, especially as the childhood uveitis service is not part of paediatric ophthalmology at some centers.

One further observation was the increase in numbers of consultations depending on the treatment escalation pathway. This is most likely explained by the time lag to escalate to TNF alpha inhibitors, as a three months waiting period is recommended to assess the effect of methotrexate [19, 26]. Treatment burden is high for children with uveitis, and multiple studies have shown the significant effect of the diagnosis and relapses on quality of life of affected children as well as their families [57, 58]. Reducing the number of consultations as well as achieving remission in a timely manner with escalating treatments sooner might potentially reduce burden for patients, their families as well as the health care facilities and increase quality of life of patients and their families. Safety and cost effectiveness would need to be further evaluated.

The major limitation of this study is its retrospective design and the low power. Non-infectious paediatric uveitis is a rare disease, presents as a mixed cohort, and treatment is mostly based on recommendations only. This cohort shows a variability in the treatments used as well as in the monitoring - as expected in real word data.

## Conclusion

Current treatment approach (steroid eye drops, followed by methotrexate, followed by TNF alpha inhibitor) for non-infectious paediatric uveitis results in good VA outcome for most patients. The treatment burden is high, especially for patients going through multiple steps of the escalation later with frequent consultations during the first year of follow up. Methotrexate is often either insufficient for disease control or causes substantial side effects. Timely treatment escalation to adalimumab might lower treatment burden for affected children, their families and health care providers.

## Data Availability

The datasets analysed during the current study are available from the corresponding author on reasonable request. Any request to share the dataset will need to be reviewed with the local ethics committee.

## Abbreviations

VA: Visual acuity
TNF: Tumor necrosis factor
SUN: Standardization of uveitis nomenclature
JIA: Juvenile idiopathic arthritis
EHR: Electronic health records
IOP: Intraocular pressure
ANA: Antinuclear antibody
HLA: Human leukocyte antigen
BASEC: Business Administration System for Ethics Committees
SD: Standard deviation
RCT: Randomized controlled trial
JAK: Janus kinase
